# Establishment and Application of a Novel Genetic Detection Panel for SNPs in Mongolian Gerbils

**DOI:** 10.3390/genes15060817

**Published:** 2024-06-20

**Authors:** Yafang Guo, Yutong Cui, Minghe Sun, Xiao Zhu, Yilang Zhang, Jing Lu, Changlong Li, Jianyi Lv, Meng Guo, Xin Liu, Zhenwen Chen, Xiaoyan Du, Xueyun Huo

**Affiliations:** 1 School of Basic Medical Sciences, Capital Medical University, Beijing 100069, China; 2 Laboratory for Clinical Medicine, Capital Medical University, Beijing 100069, China; 3 Beijing Key Laboratory of Cancer Invasion and Metastasis Research, Beijing 100069, China

**Keywords:** Mongolian gerbils, SNP, genetic quality control, second-generation sequencing

## Abstract

The Mongolian gerbil is a distinctive experimental animal in China, as its genetic qualities possess significant value in the field of medical biology research. Here, we aimed to establish an economical and efficient panel for genetic quality detection in Mongolian gerbils using single-nucleotide polymorphism (SNP) markers. To search for SNPs, we conducted whole-genome sequencing (WGS) in 40 Mongolian gerbils from outbred populations. Reliable screening criteria were established to preliminarily select SNPs with a wide genome distribution and high levels of polymorphism. Subsequently, a multiple-target regional capture detection system based on second-generation sequencing was developed for SNP genotyping. Based on the results of WGS, 219 SNPs were preliminarily selected, and they were established and optimized in a multiple-amplification system that included 206 SNP loci by genotyping three outbred populations. PopGen.32 analysis revealed that the average effective allele number, Shannon index, observed heterozygosity, expected heterozygosity, average heterozygosity, polymorphism information content, and other population genetic parameters of the Capital Medical University (CMU) gerbils were the highest, followed by those of Zhejiang gerbils and Dalian gerbils. Through scientific screening and optimization, we successfully established a novel, robust, and cost-effective genetic detection system for Mongolian gerbils by utilizing SNP markers for the first time.

## 1. Introduction

The Mongolian gerbil is a distinctive experimental animal in China, and it possesses significant value in the field of medical biology research. With the Mongolian gerbil, studies have been conducted on variations in the Circle of Willis (CoW) [1,2,3], cerebral ischemia [4,5,6], and gastric diseases associated with *Helicobacter pylori* [7,8]. Additionally, gerbil stroke models [9,10,11,12] and hereditary spontaneous diabetes models [13,14] have been established. Genetic monitoring is the main method employed to assess genetic diversity and the stability of the genetic background in experimental animals, enabling the detection of potential genetic mutations and genetic pollution [15]. Whole-genome sequencing (WGS) has already been conducted for the Mongolian gerbil [16], and the size of its whole genome length is about 3,931,855,312 bp [17]. Unfortunately, the available genetic testing methods for Mongolian gerbils are extremely limited. At present, the prevailing method of genetic analysis for gerbils in China is based on microsatellites [18,19,20,21], but an internationally acceptable testing approach for genetic quality remains absent.

In comparison with microsatellites, single-nucleotide polymorphisms (SNPs) possess the advantages of widespread distribution, enhanced stability, simplified analysis, and high throughput capacity, rendering them more suitable as markers for genetic monitoring. SNP markers have been widely used in genetic evaluations in many kinds of laboratory animals, such as mice [22,23,24,25] and rats [26,27,28,29]. They have been widely used in animal and plant breeding, disease-resistance gene markers, and the screening and identification of varieties of disease-related genes. The existing standard GB14923-2022 [30] “Genetic Quality Control of Experimental Animals” (China) recommends the utilization of 35 SNP marker genes for the genetic monitoring of inbred mice; however, there is currently a lack of well-defined SNP-based genetic quality assessment methods for Mongolian gerbils. Therefore, it is important to establish an internationally advanced genetic quality testing method using SNPs for Mongolian gerbils. This not only fills the gap in relevant standards but also holds significant implications for the enhancement of the genetic testing technology level and ensuring the overall quality of the Mongolian gerbil.

In this study, we succeeded in screening SNP loci with a wide distribution across the genome, high polymorphism rates, no linkage disequilibrium, and high confidence using large-scale whole-gene resequencing and multiple-target capture techniques. We developed an SNP genotyping method suitable for high-throughput genetic detection in Mongolian gerbils.

## 2. Materials and Methods

### 2.1. Animal Samples

Mongolian gerbils were collected from three outbred populations from Zhejiang (*n* = 37), CMU (*n* = 31), and Dalian (*n* = 32) and two inbred populations with diabetes (*n* = 10) and cerebral ischemia (*n* = 10). The gender of the gerbils was randomly selected. We randomly selected animals from the inbred populations and selected distantly genetically related individuals from the outbred populations. The original animals in the populations of Zhejiang, CMU, and Dalian were captured in inner Mongolia in 1978, 1982, and 1972, respectively. This study was approved by the Animal Experiments and Experimental Animal Welfare Committee of Capital Medical University (AEEI-2021-309).

### 2.2. DNA Extraction

A DNA extraction kit (FastPure^®^ Cell/Tissue DNA Isolation Mini Kit, Vazyme Biotech Co., Ltd., Nanjing, China) was used to extract genomic DNA from the Mongolian gerbils’ ear and tail tissues. The concentration and purity of DNA from each sample were determined with an A260/A280 measurement using a Nanodrop 2000C micro-spectrophotometer (Thermo Fisher Scientific Inc., Waltham, MA USA) and further evaluated through agarose gel electrophoresis; then, the samples were stored at −20 °C.

### 2.3. Sequencing and Quality Control

Due to the limited availability of a comprehensive SNP database for the Mongolian gerbil, we conducted whole-genome sequencing (WGS) with a coverage of 10× on 20 Mongolian gerbils from Zhejiang and 20 CMU Mongolian gerbils, aiming to economically seek polymorphic SNP loci among animals through sequence comparison. Genomic DNA was sequenced with the Combinatorial Probe–Anchor Synthesis method in the DNBSEQ System (company, Beijing GenePlus Clinical Laboratory Co., Ltd., Beijing, China; machine, DNBSEQ-T7RS; read lengths, PE150; date out, 1.75-7T). Raw reads were filtered using the fastp-v0.21.0 software (https://github.com/OpenGene/fastp, accessed on 16 January 2023). Paired-end reads were mapped to MunDraft-v1.0 (https://www.ncbi.nlm.nih.gov/genome/gdv?org=meriones-unguiculatus&group=muridae, accessed on 16 January 2023) using the BWA-v0.7.17 software (https://sourceforge.net/projects/bio-bwa/files/, accessed on 16 January 2023). SNP calling and filtering were performed with the GATK-v4.0.0.0 software (https://gatk.broadinstitute.org, accessed on 16 January 2023). The BCFtools-v1.20-6 software (http://www.htslib.org/download/, accessed on 16 January 2023) was used for quality control of the data, and the following standards were set: (i) removal of SNP loci with a QualByDepth value of less than 2.0; (ii) removal of SNP loci with a FisherStrand value of less than 60.0; (iii) removal of SNP loci with an RMSMappingQuality value of less than 30.0; and (iv) removal of SNP loci with a sequencing depth of less than 8.0.

### 2.4. SNP Locus Selection

Our SNP screening criteria included the following five points: (i) for a uniform distribution across the genome, candidate loci were selected from different fragments to avoid linkage; (ii) the genotype frequency ranged between 25% and 75%, which was to ensure the polymorphism of the loci; (iii) to improve the amplification efficiency, no other SNPs or indels were found within a 200 bp region upstream or downstream of the selected loci; (iv) no complete linkage was observed among the selected loci; and, additionally, (v) so that they would correlate as much as possible with the phenotype, these SNPs predominantly resided in gene regions with a high concentration of missense mutations, nonsense mutations, and nonstop mutations. In accordance with this procedure, we screened a high-quality SNP locus panel.

### 2.5. Primer Design and Sequencing

After obtaining the candidate SNPs, we handed over the region of interest (ROI), that is, the information on the physical locations of the SNPs, to iGeneTech Biotechnology Beijing Co. Ltd., Beijing, China, and used MFEprimer-v3.1 to design and validate multiple PCR primers that targeted the genomic sequence of the SNPs in our panel. Based on the thermodynamic stability [31,32], highly specific multiplex primers were designed on both sides of the ROI; the amplicon was 160–260 bp. We then evaluated primer dimerization and non-specific amplification, tested the designed and synthesized primers, and replaced the primers that had a poor detection effect. Subsequently, sequencing was performed on an Illumina^®^ NovaSeqTM 6000 system (Illumina, Inc., San Diego, CA USA) using amplicon-targeted capture in the PE150 paired-end sequencing mode.

### 2.6. Population Genetic Analysis

The genotype of each SNP locus in the form of AA, BB, and AB for all samples was input into the Popgen.32 Analysis software [33]. We used this software to calculate population genetic parameters such as average effective allele number, Shannon index, observed heterozygosity, expected heterozygosity, and average heterozygosity for different individuals at each SNP locus.

### 2.7. Compilation of Principal Component Analyses and Population Structure

Principal component analysis (PCA) and R (v4.3.2) were used to generate a PCA figure and a 3D PCA image. To estimate the genetic structure of our dataset, STRUCTURE v2.3.4 (https://web.stanford.edu/group/pritchardlab/structure.html, accessed on 16 January 2023) was run with 10,000 burn-ins and 50,000 iterations for the Markov Chain Monte Carlo (MCMC) method [34,35]. The frequency model of ‘correlated allele frequencies’ among populations was used, and an ‘admixture’ with K values ranging from 2 to 6 was tested across a total of ten runs. The estimated ln probability values (−LnP(D)) for each K value were plotted using the online STRUCTURE harvester tool v0.6.94 [35] (http://alumni.soe.ucsc.edu/~dearl/software/structureHarvester/, accessed on 16 January 2023).

## 3. Results

### 3.1. Establishment and Optimization of the SNP Detection System

**Figure 1:**
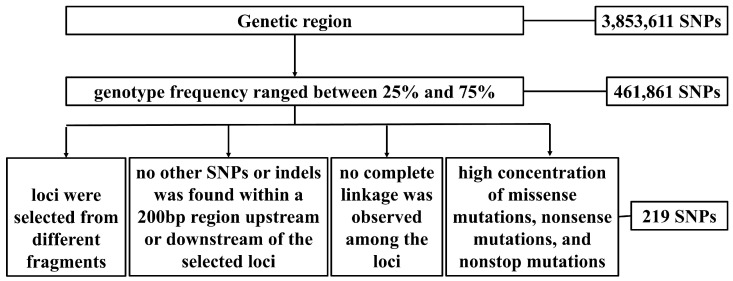
SNP screening criteria.

By conducting a comparative analysis of the WGS results for 40 Mongolian gerbils, 3,853,611 SNP loci were identified in the genetic region. To screen an efficient SNP panel for genetic quality control in Mongolian gerbils, we established criteria for selecting high-quality SNPs from the SNP dataset derived from WGS (Figure 1). Based on the SNP screening criteria, we selected 219 high-quality loci for the development of the Mongolian gerbil SNP detection system (Supplementary Table S1).

In order to further optimize the effective SNPs, we genotyped 219 SNP loci in 120 samples from five Mongolian gerbil populations. We deleted thirteen loci for the following reasons: (1) MAPQ < 59, indicating that the reads located at these loci were not unique compared with the reference genome and that the genotyping results were unreliable; (2) the loci were homomorphic in all samples; (3) there was complete linkage (Supplementary Table S2). In summary, a panel of 206 high-quality SNP loci was optimized and recommended for the genetic evaluation of Mongolian gerbil populations (Supplementary Table S3).

### 3.2. Genetic Analysis of the Outbred Mongolian Gerbil Populations

We applied the SNP detection system to the analysis of the genetic structure of three outbred Mongolian gerbil populations using the Popgen.32 software (Table 1). Our findings revealed that the Mongolian gerbils from CMU exhibited the highest values for various parameters, including the average effective allele numbers (ne), Shannon index (I), observed heterozygosity (Obs Het), expected heterozygosity (Exp Het), and average heterozygosity (Ave Het). The gerbils in the Zhejiang, diabetes, Dalian, and cerebral ischemia populations displayed progressively lower values.

**Figure 2:**
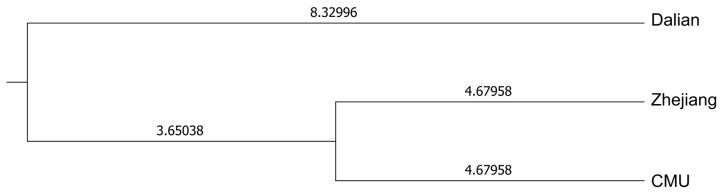
Dendrogram based on Nei’s genetic distance and the UPGMA method. The numbers on each branch represent the length of each branch.

A dendrogram based on Nei’s genetic distance and the UPGMA method was created for the three outbred populations (CMU, Zhejiang, and Dalian), revealing that the Dalian Mongolian gerbils exhibited the greatest genetic divergence from the other Mongolian gerbil populations. Conversely, the Zhejiang population displayed a closer genetic distance to the CMU population (Figure 2).

### 3.3. Structure Analysis of the Three Mongolian Gerbil Populations

**Figure 3:**
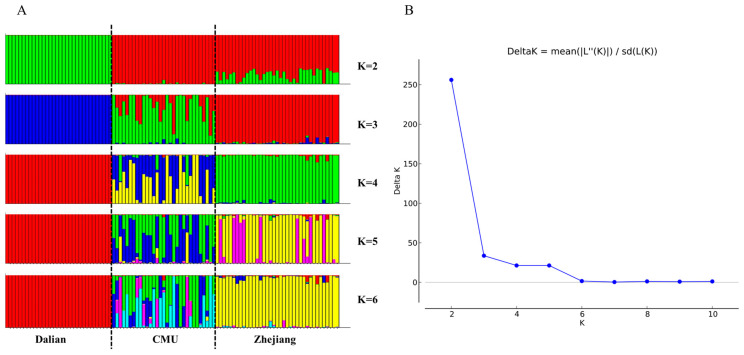
Structure analysis of 206 SNPs in the three populations: (**A**) bar plot of K values ranging from 2 to 6; (**B**) the distribution of K values with ΔK.

The results of the population genetic structure analysis are shown in Figure 3. The number of assumed populations (K) was set to 2–6 in this study. It was found that the ΔK value was highest at K = 2, and it was recommended to divide this into two populations. The Dalian populations were separated from the other populations at K = 2. This was consistent with the results of the genetic distance analysis. Coherently, we found that the three populations showed different proportions of components at K = 3. These data indicate that the genetic structure of the outbred CMU population was similar to that of the outbred Zhejiang population.

### 3.4. Principal Component Analysis in the Three Mongolian Gerbil Populations

**Figure 4:**
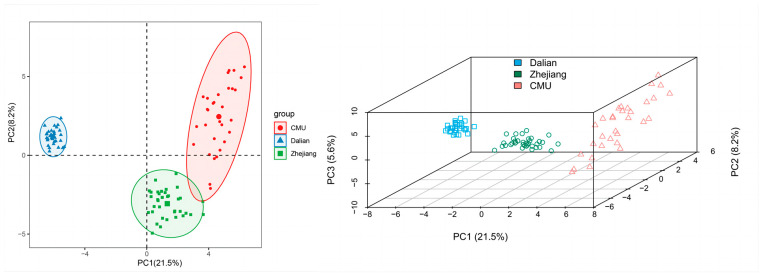
The plot of the two-dimensional (**A**) and three-dimensional (**B**) principal component analyses among the three populations with 206 SNPs.

It is well established that PCA can be used to show the main differences in genetic distances between complex samples through a reduction in the data dimensionality. The results of PCA based on the allele frequency distributions of 206 polymorphic SNP loci in all three outbred populations are shown in Figure 4. In the genotype-based PCA results, PC1 (21.5%) and PC2 (8.2%) extracted 29.7% of the total genetic variation. The principal component analysis completely separated the Dalian population in a two-dimensional schematic diagram of PC1 (Figure 4A). All populations were separated by three PCs (Figure 4B). Again, the results of the PCA demonstrated that the Zhejiang and CMU populations were closer in terms of genetic distance.

### 3.5. Genetic Analysis of the Outbred Mongolian Gerbil Populations

We also observed that the genotypic distribution of some loci was population-specific, and there were significant differences between populations. For instance, the locus MG15 exhibited polymorphism in the Dalian gerbils and Zhejiang gerbils with genotypes AG/GG/AA, but it exhibited monomorphism in the CMU gerbils, with only one kind of genotype (GG). The locus MG126 exhibited homomorphism in the Dalian and CMU gerbils, with only one genotype (CC or TT, respectively), whereas the Zhejiang gerbils exhibited three genotypes: TT/TC/CC. The three outbred populations could be easily distinguished with these two loci (Table 2). In total, we found seven loci that were specific in different populations (Table 2); they could be regarded as unique loci of a certain population and applied to easily distinguish the three outbred populations.

### 3.6. Genetic Analysis of the Inbred Mongolian Gerbil Lines

In the panel of 206 SNP loci, 156 were found to be monomorphic in the inbred cerebral ischemia and 77 loci were found to be monomorphic in the inbred diabetes lines; 64 loci were monomorphic in both lines, but 15 of them exhibited different genotypes between the two lines and could be used to distinguish the two inbred populations (Table 3).

## 4. Discussion

This study presents the pioneering utilization of WGS to identify and select SNPs in Mongolian gerbils. By implementing stringent criteria, from a genetic region with 3,853,611 SNPs, we successfully identified 219 high-quality SNP loci with high levels of polymorphism and wide distribution across the genome and subsequently developed a multiple-SNP detection system.

In comparison with single-SNP amplification and genotyping methods, the multiple-target region capture technology (MultipSeq Primer for the genotyping of SNP loci) based on next-generation sequencing technology is more cost-effective and has higher efficiency, enabling the simultaneous acquisition of a greater number of loci and genotyping results [32,36,37]. In the present study, by testing the 219 selected SNPs in 120 animals, it was found that three loci had a low amplification quality, seven loci presented the same genotype in all animals, and three loci were completely linked with other loci. These loci were omitted due to their low capabilities for distinguishing different gerbil populations.

The use of reasonable criteria in the selection of high-quality SNPs that are suitable for genetic detection is a critically important issue. We noted that the loci were on separate chromosomes or sufficiently far apart on the same chromosome to ensure a wide distribution and show minimal linkage disequilibrium [38,39,40]. First, candidate loci were selected from different fragments across the entire genome. Second, no complete linkage was observed among the loci, thus improving the detection efficiency. To ensure that the loci were SNPs rather than SNVs, SNP sites were only conserved if MAF > 0.3 [40]. Third, the genotype frequency ranged between 25% and 75% in this study. To ensure the specific amplification of each locus [41], Wang et al. compared the reference genome of grapes and selected SNP loci with no other variations within the range of 100 bp before and after [40]. Fourth, no other SNPs or indels were found within a 200 bp region upstream or downstream of the selected loci in this study, thus further ensuring the specificity of locus amplification. SNPs were preferably located in coding regions [41], ensuring their close association with phenotypic changes. Fifth, in this study, the SNPs predominantly resided in gene regions with a high concentration of missense mutations, nonsense mutations, and nonstop mutations. These strict screening criteria were reasonable and creative and ensured the high reliability, polymorphism, and even distribution of all selected loci.

The average effective heterozygosity is an important parameter of population genetic diversity, effectively reflecting the richness of the identified genes [15,33]. When STR is employed as a marker for the detection of genetic diversity, it is generally believed that the population genetic diversity is low when the average effective heterozygosity is less than 0.5. When the average effective heterozygosity of a population is greater than 0.7, the genetic diversity is high [15]. However, the absolute values of the average effective heterozygosity of the three populations of gerbils in the present study were all lower than 0.5 [33,42]. This may be attributed to the inherent characteristics of different genetic markers; STR is always multi-allelic, and the number of alleles at a single locus can even reach higher than 10, while SNPs usually have only two alleles, indicating that we should apply different criteria to evaluate the population genetic diversity when using different markers [33]. Thus, our data indicated good genetic diversity among the three outbred populations. The results showed that the CMU population exhibited the highest genetic diversity; the Dalian group, on the other hand, exhibited the lowest values of genetic heterozygosity, which may have been related to the time at which the original animals were captured from the wild; the longer the duration of their confinement, the greater the reduction in their genetic diversity.

The loci exhibiting population-specific genotype distributions can serve as highly informative markers for population identification. At present, the existing standard GB14923-2022 [30] “Genetic Quality Control of Experimental Animals” (China) recommends the utilization of 35 SNP marker genes for the genetic monitoring of four inbred strains of mice. Wei et al. [43] also developed a combination of eight SNP loci that can distinguish 10 inbred mice strains. In the present study, seven SNP loci could be regarded as unique loci of a certain population and applied to easily distinguish the three outbred populations. The MG15 locus may be used to effectively differentiate the CMU population from the Dalian and Zhejiang populations, while a single locus, MG126, may be used to distinguish between the three populations of CMU, Zhejiang, and Dalian. A combination of fifteen SNP loci may be used to distinguish the two inbred populations.

Compared to the STR detection method, STR detection has advantages such as simple operation, easy interpretation, and low cost. The mutation rate of SNPs is only one-thousandth of STR, which is more stable during population inheritance. However, there are certain limitations of this study. The population cultivation of Mongolian gerbils is not as mature as that of mice. Only three populations were collected in the outbred population, and the number of animals selected for sequencing validation was small, resulting in a scarce amount of data. Some differential loci need to be further determined by expanding the sample size of the population in later stages of research.

## 5. Conclusions

We successfully established a novel genetic detection system for SNPs in Mongolian gerbils that is efficient and precise when conducting genetic analyses on several outbred and inbred gerbil populations. These findings will significantly advance the level of genetic detection for characteristic gerbils in China.

## Figures and Tables

**Table 1:** Genetic structure of the three outbred Mongolian gerbil populations.

Mongolian Gerbils	Na	ne	I	Obs Hom	Obs Het	Exp Hom	Exp Het	Ave Het	Percentage of Polymorphic Loci
CMU	1.9903	1.6953	0.5753	0.6019	0.3981	0.6002	0.3998	0.3934	99.03%
Zhejiang	2.0000	1.6550	0.5594	0.6229	0.3771	0.6167	0.3833	0.3782	100%
Dalian	1.5146	1.3408	0.2882	0.7770	0.2230	0.8014	0.1986	0.1955	51.46%

Note: Na: average allele numbers; Ne: effective allele numbers; I: Shannon index; Obs Hom: observed homozygosity; Obs Het: observed heterozygosity; Exp Hom: expected homozygosity; Exp Het: expected heterozygosity; Ave Het: average heterozygosity.

**Table 2:** Allele frequencies of seven SNP loci in the three outbred populations.

Number	Genotyping	Zhejiang	Dalian	CMU	*p* Value
MG15	AA	0.08	0.09	0.00	<0.001
	AG	0.35	0.47	0.00	
	GG	0.57	0.44	1.00	
MG89	CC	0.00	0.00	0.68	<0.001
	CT	0.05	0.00	0.32	
	TT	0.95	1.00	0.00	
MG126	CC	0.03	1.00	0.00	<0.001
	CT	0.09	0.00	0.00	
	TT	0.89	0.00	1.00	
MG159	CC	0.97	0.59	0.97	<0.001
	TT	0.03	0.41	0.03	
MG164	AA	0.32	0.97	0.74	<0.001
	AC	0.35	0.03	0.26	
	CC	0.32	0.00	0.00	
MG206	AA	0.22	0.97	0.39	<0.001
	AG	0.62	0.03	0.42	
	GG	0.16	0.00	0.19	
MG215	CC	0.03	0.00	0.90	<0.001
	CT	0.54	0.00	0.10	
	TT	0.43	1.00	0.00	

**Table 3:** Fifteen loci with different phenotypes between the two inbred lines.

Number	CHROM	POS	Variant_Classification	REF	ALT	Inbred Cerebral Ischemia Line	Inbred Diabetes Line
MG37	NW_018657859.1	1,081,095	5′Flank	C	G	G/G	C/C
MG46	NW_018657888.1	735,326	Missense_Mutation	A	G	A/A	G/G
MG47	NW_018657888.1	1,469,235	Nonstop_Mutation	A	T	T/T	A/A
MG49	NW_018657893.1	1,416,535	Missense_Mutation	C	T	C/C	T/T
MG54	NW_018657915.1	154,939	Intron	T	C	T/T	C/C
MG73	NW_018658000.1	110,223	Missense_Mutation	T	C	T/T	C/C
MG82	NW_018658044.1	1,083,386	Missense_Mutation	G	T	T/T	G/G
MG94	NW_018658087.1	982,880	Missense_Mutation	T	C	C/C	T/T
MG107	NW_018658151.1	621,718	3′Flank	G	T	T/T	G/G
MG114	NW_018658168.1	333,441	5′Flank	C	T	T/T	C/C
MG124	NW_018658203.1	697,270	Missense_Mutation	C	T	T/T	C/C
MG132	NW_018658278.1	540,596	Nonsense_Mutation	C	A	A/A	C/C
MG142	NW_018658707.1	206,847	Missense_Mutation	C	A	A/A	C/C
MG190	NW_018661696.1	2303	Missense_Mutation	A	G	G/G	A/A
MG219	NW_018692212.1	1211	5′Flank	G	A	G/G	A/A

## Data Availability

The datasets used and/or analyzed during the current study are available from the corresponding author upon reasonable request.

## Supplementary Materials

The following supporting information can be downloaded at https://www.mdpi.com/article/10.3390/genes15060817/s1: Table S1: Panel with 219 SNPs from Mongolian gerbils; Table S2: The thirteen SNP loci that were deleted; Table S3: Panel with 206 SNPs from Mongolian gerbils; Table S4: Mongolian gerbil primers; Table S5: PCA software codes for analysis.
